# Sociocultural and individual determinants for motivation of sexual and reproductive health workers in Papua New Guinea and their implications for male circumcision as an HIV prevention strategy

**DOI:** 10.1186/1478-4491-11-7

**Published:** 2013-02-19

**Authors:** Anna Tynan, Andrew Vallely, Angela Kelly, Martha Kupul, James Neo, Richard Naketrumb, Herick Aeno, Greg Law, John Milan, Peter Siba, John Kaldor, Peter S Hill

**Affiliations:** 1 Australian Centre for International & Tropical Health, School of Population Health, University of Queensland, Herston Road, Herston, Queensland, 4006, Australia; 2 Public Health Interventions Research Group, Kirby Institute, University of New South Wales, Cliffbrook Campus, 45 Beach Street, Coogee, New South Wales, 2034, Australia; 3 Sexual & Reproductive Health Unit, Papua New Guinea Institute of Medical Research, P.O. Box 60, Goroka, Eastern Highlands Province, 441, Papua New Guinea; 4 International HIV Research Group, School of Public Health and Community Medicine, University of New South Wales, High Street, Kensington, 2052, Australia; 5 Sexual Health and Disease Control Branch, National Department of Health, P.O. Box 807, Waigani, National Capital District, 131, Papua New Guinea

## Abstract

**Background:**

The motivation of health workers (HWs) to deliver services in developing countries has been described as a critical factor in the success of health systems in implementing programmes. How the sociocultural context of Papua New Guinea (PNG) affects the values, motivation and actions of HWs involved in sexual and reproductive health services is important for policy development and programme planning. With interest in male circumcision (MC) as an HIV prevention option in PNG, this study explored the perceptions and motivations of HWs involved in sexual and reproductive health services in PNG, examining their implications for the possible future roll out of a national MC programme.

**Methods:**

A multi-method qualitative study was conducted with HWs across a range of health care professions working in sexual health facilities. A total of 29 in-depth interviews and one focus group discussion were completed. Qualitative thematic analysis of the transcripts and field notes was undertaken using a social constructivist approach and complemented by documentary organizational, programme and policy analysis.

**Results and discussions:**

Introduction of new health programmes, such as a MC programme for HIV prevention, are likely to impact upon one or more of the many motivational determinants. Social–cultural and individual factors influencing HW motivation to be involved in sexual and reproductive health services in PNG included community expectation and concern, sense of accomplishment and religious conviction. Strong links to community responsibility outweighed organizational ties. Faced with an often dysfunctional work environment, HWs perceived themselves as responsible to compensate for the failed health system. The impact of community influence and expectation needs to be considered when introducing a MC programme, particularly to communities in PNG where penile foreskin cutting is a common and accepted practice.

**Conclusions:**

The potential contribution to the success of a MC programme that HWs may have means that taking into account the differing needs of communities as well as the motivational influences on HWs that exist within the sociocultural environment is important. These findings will assist not only in programme planning for MC, but also in the expansion of other existing sexual and reproductive health services.

## Background

Following three clinical trials in Africa which showed that male circumcision (MC) reduces the risk of HIV acquisition for men during vaginal intercourse by up to 60%, UNAIDS and the World Health Organization (WHO) has recommended that MC be considered as an essential component of comprehensive HIV prevention programmes in high-prevalence settings [1-5]. There has been interest in aligning MC with other HIV intervention programmes in Papua New Guinea (PNG), a country of significant geographical, linguistic and cultural diversity where different forms of penile cutting appear to be common in some communities [6-9]. PNG has amongst the highest HIV prevalence in the Asia Pacific Region and the epidemic is primarily linked to heterosexual transmission exhibiting substantial geographic heterogeneity, with cases clustered in a number of key provinces [10-15]. A country-specific mathematical model suggests that MC could have a significant impact on the HIV epidemic in PNG but that increasing condom use and early initiation of antiretroviral therapy could potentially have a greater impact [10]. Difficulties have already been described in the capacity of the health system to undertake even simple health programmes with demonstrated challenges in governance, financing, access to equipment and staffing [16,17]. Complex interaction of the sociocultural environment and the health system has also been shown with evidence of health workers (HWs) already participating informally and formally in penile cutting activities, although no national programme has yet been established [18-20]. Understanding the social and individual factors influencing sexual and reproductive HW motivation to engage in sexual and reproductive services and the impact this may have on more complex intervention programmes such as medical MC is critical.

The purpose of this paper is to identify the sociocultural and individual factors that motivate frontline HWs, including medical doctors, nursing officers, community health workers (CHWs), health extension officers, counsellors and other fieldworkers, to become active participants in improving sexual and reproductive health outcomes in their communities. In particular, the paper aims to explore HWs’ experience of and performance in sexual and reproductive health services in PNG and how this may impact on a potential future MC programme if deemed appropriate. This research forms a component of a wider multi-disciplinary, community-based research programme to investigate the acceptability, epidemiological impact, cost-effectiveness and potential service delivery model options for programme implementation of MC in PNG, commonly referred to as the Male Circumcision Acceptability and Impact Study. This study was carried out by the PNG Institute of Medical Research in collaboration with the University of Queensland and the University of New South Wales.

### Health worker motivation in the context of HIV: implications for a male circumcision programme in PNG

The motivation of HWs can potentially affect the provision of health services and, according to the World Health Report 2006, a capable and motivated health workforce is required to achieve any of the Millennium Development Goals, including universal access to HIV prevention and treatment [21-24]. In the context of HIV, HW motivation to engage in HIV services are influenced by HIV-related challenges including the additional strain on health service provision due to the burden of the epidemic in some regions, fears of infection, and a discriminatory attitude among HWs towards HIV due to dealing with matters pertaining to sexual behaviours [14,25,26]. HW motivation may also be influenced by the disproportionate funding, resourcing, infrastructure and historical focus on HIV that have been described in some contexts [27]. Motivation of HWs in developing countries to engage in recently introduced programmes and practices has also been highlighted in the literature to be an important component of programme success [28,29]. Given the complexity of rolling out adult MC programmes that has already been described in Africa, understanding HW motivation in the context of sexual reproductive health services and application to MC programme roll-out is important for policy development.

Effective engagement of human resources has been a widely discussed topic in the implantation of a MC programme in African countries [30-32]. Significant numbers of clinicians, counsellors and support staff are needed to implement even a modest programme, given the relative technical difficulty of the surgery compared with other prevention programmes such as immunization [33,34]. In some African countries, task shifting, or the delegation of surgical steps to a trained non-physician clinician such as a nurse or clinical officer, has been utilized to greatly expand the size of the workforce available; whilst in other countries, non-physicians are restricted from performing MC due to the countries’ regulatory and legal frameworks [33]. Previous research in PNG has shown that non-physician clinicians including CHWs have been called upon to implement no-scalpel vasectomy (NSV) programmes and may be considered for a MC programme [35]. Suggested roll-out strategies for a MC programme that may be relevant to PNG include integrating the programme into exiting services or setting up centres of excellence based in regional areas with satellite clinics attached [36]. Whatever the decision, understanding how HWs are likely to engage in the MC programme should the government of PNG decide to proceed with such a policy in the future is important, particularly as evidence also exists of all levels of HWs being involved in penile foreskin cutting activities in PNG [37].

Previous research has highlighted the complex and diverse nature of penile foreskin cutting practices in PNG [6,7,9]. Practices have been described to include traditional practices that are embedded in customary rituals; contemporary practices that are not associated with customary observations but are an outcome of the influence of peers and the sociocultural environment; and medical MC [6-8]. Adult penile foreskin cutting appears to be common in many parts of PNG within various sociocultural contexts, and men from a variety of different settings in PNG have been shown to be notionally supportive of MC for HIV prevention [6-8]. A survey among 869 men in National Capital District (NCD), Madang Province, Enga Province and Oro Province found that 47% had a longitudinal cut or dorsal slit (foreskin cut but not removed) and 10% a circumferential cut (complete removal of foreskin) [38]. HWs have been described to be involved in management of complications following penile cuts completed by non-HWs and the unauthorized involvement of HWs in penile cutting activities in various contemporary and cultural penile cutting activities [6,8]. Other research has also shown that of the 396 men reporting a longitudinal foreskin incision, 15% advised that it had been completed by a PNG HW outside any formal health programme [38]. With this in mind, there is likely to be a number of different challenges across PNG in the implementation and sustainability of a MC programme, and specific understanding about motivation for HWs to engage in sexual and reproductive health programmes and how this may impact the delivery of a MC programme in PNG is essential.

### Framework for understanding health worker motivation

**Figure 1:**
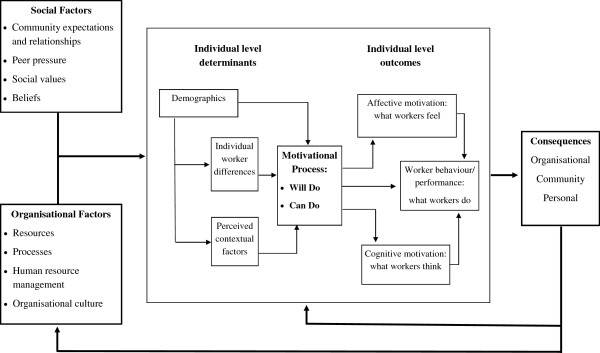
**Framework for determinants of health worker motivation **[40].

HW motivation is complex and there are multiple factors that influence HWs’ willingness to apply themselves to their tasks and be successful in delivering health services [39-41]. Individual HW performance relates to competencies and resource availability; however, motivation to deliver health services is also integral to performance and is underpinned by the organizational structure, the sociocultural environment and individual characteristics of the HWs [39,40,42]. Most research on HW motivation has occurred in high-income countries, whereas little attention has been given to HW motivation in developing countries [25,41-47]. The framework developed by Franco, Bennet and Kanfer (Figure 1) is one framework that has been applied to low-income and middle-income country settings [39,40]. Synthesized from the motivation literature from developed countries, this framework describes the complex transaction between individuals and their work environment and the impact this has on motivation [39,40]. HW motivation is described not as an attribute of the individual, but rather as a result of the transaction between organizational factors (organizational culture, support structures, resources and processes), social factors (community expectations, social values and peer pressure) and the individual [39,40]. Drawing on this conceptual framework, this study examined the broad range of HW motivational determinants underpinned by the unique sociocultural context of PNG.

### Organizational factors

Organizational factors thought to have an effect on motivation include resource availability; organizational structures and culture; efficiency of process; and human resource management practice [39]. Health-sector reform has the most potential to have an impact on organizational factors of motivation, and strategies for this have largely been covered in the literature [40-42]. Organizations can influence HW motivation through efforts to improve worker proficiency with processes and resources and efforts to improve work culture [39]. Factors such as shortage of supplies and poor access to development and training can affect quality of care and motivation of the HW [26]. A range of organizational incentives have been described to assist with motivation and retention of HWs in developing countries and include improved working and living conditions, continuing education, training and professional development, and improved supervision and management [21,40-42,44].

Recent research into factors influencing rural HW performance in PNG showed the importance of organizational determinants such as work climate, good leadership, supervision and access to resources as important contributors to increased performance [18,19]. However, while structural and organizational determinants of motivation have been addressed, little research has been conducted with regard to individual and sociocultural determinants of HW motivation to deliver services, particularly in the context of HIV prevention and treatment programmes [26]. As Franco, Bennet, Kanfer and Stubblebine argue, further exploration of social and individual determinants is necessary to understand the motivational context of HWs working in developing and transitioning countries due to the vast social–cultural differences and potential impact on organization [40]. Country-specific data on social and individual determinants of HW motivation are therefore integral to understanding the complexities of working within sexual and reproductive health services, particularly in diverse settings such as PNG.

### Social factors

Social factors, or cultural and community influences, are described by the framework as distal determinants of motivation [39,40]. These social factors affect the relative importance of the different determinants of motivation and the relationships between them, and potential impact on the provision of health services particularly for those working in the HIV and AIDS [27]. For example, HW performance has potential to be influenced by the high level of donor attention on HIV prevention and care; community expectations of how services should be delivered; and the personal interactions HW have with clients [26,39]. The need to tailor programmes to more fully consider the local workforce and local culture is integral to address motivational determinants for the needs of specific groups within each context [20,26,40].

Christianity is well established in PNG with over 90% of the population belonging to a Christian denomination [45,46]. The churches provide roughly 50% of PNG’s education and health services; the influence of Christianity also extends to government services, however, with most government HWs identifying as Christian [46,47]. The role of religious faith inspires a need to do good and take care of those who are suffering and has been described as ‘a culture of service’ that influences the practices of HWs in PNG [18]. The impact that Christian frameworks have on sexual and reproductive health service delivery in PNG has been explored with both potential positive impacts (e.g., greater adherence to antiretroviral therapy for those attending church facilities) [45,47-49] and negative impacts (e.g., the reluctance of staff to give out condoms due to their religious beliefs) [50-53].

The relationships between health systems, the HW and the community have also been described as a key driver of HW performance particularly in rural areas of PNG [18,20]. Furthermore, stronger relationships between communities and HWs have been shown to result in better outcomes in health promotion projects in PNG [54]. If MC was to be introduced at national or sub-national level in PNG, understanding how the social environment influences motivation for the provision of sexual and reproductive health services will be critical to future public health policy formulation.

### Individual processes for motivation

Within these larger organizational and societal contexts, HW motivation is an individual, internal and unobservable process [40]. Understanding individual HWs’ experiences and the ways in which they interpret their reality provides insight into their willingness to respond to certain interventions [21]. According to Franco, Bennet, Kanfer and Stubblebine’s model, individual level determinants can be categorized into demographic characteristics, individual differences (personality, self-concept, individual goals, value systems and expectations and experience of outcomes) and perceived contextual factors such as perception of work environment coupled with the individual workers’ technical and intellectual capacity to perform key tasks within available resources (Figure 1) [26,39].

The outcomes of these determinants, conceptualized by the HW, result in behavioural, emotional and cognitive responses, with the relative weight of the response dictated by the HW [26,40]. For example, understaffing that results in excessive workload and delegation of duties beyond the position description of the worker has negative impacts on HW performance and has been described as one of the HIV services related challenges on HW motivation [26]. HW density in PNG is comparably lower than other Asia Pacific countries [42]. Despite the potential for increased workload, rural HWs in PNG have been shown to acknowledge the positive aspects of additional skills and the autonomy over their role that understaffing can create [18].

The importance of understanding HWs’ relationships to the health system, resources and environment in which they operate should not be underestimated as HW motivation to engage in programmes can play an essential role in delivering services [39]. In addition to community acceptability and epidemiological impact, individual-level relationships, incentives, meanings and potential attitudes of HW within the milieu of HIV prevention needs to be examined in PNG before further discussions about a possible MC programme. Research agendas suggested by WHO and other advisors have not considered the potential impact HW motivation will have on an MC programme or how HW motivation may result in service enhancements, independent of structural change [54,55]. HW motivation is a complex process and this paper will discuss the many layers of influences upon HWs working with sexual and reproductive health service related challenges, in particular the individual and social-level determinants. Organizational determinants for successful sexual and reproductive health services including the need to strengthen leadership, improve resources and effectiveness of services have been explored in great detail in previous studies of the PNG health system and thus will not be examined directly here [16]. By drawing attention to this broad range of influences of social and individual determinants, this paper aims to help policy-makers view the complexity of HW worker motivation in PNG within the current health system environment and how this may impact on a MC programme for HIV prevention.

## Methods

### Theoretical framework and study design

Using a multi-method qualitative approach, the research team conducted an analysis of selected programmes within the PNG health system to examine human resource performance and motivation to work in the sexual and reproductive health field. To assist with identifying individual and sociocultural determinants of motivation, a social constructivist approach was utilized. Social constructivism aims to understand how individuals construct their own realities and meanings utilizing their culturally available language and subjective experience to make sense of their daily lives [21,56]. Individuals do not operate as isolated entities but rather are influenced by processes within communities that influence what is known, important or understood [57]. Motives are the joint result of personal history, subjective experience, social norms about appropriate behaviours and reasons to act, and available agendas [20]. Guided by this approach, the study sought to investigate the sociocultural and individualized factors most salient in shaping the motivation of HWs in the provision of sexual and reproductive health services in PNG and how this may affect a MC programme if it is introduced.

The data collection was completed over two phases from 2009 until 2011. Phase 1 fieldwork provided the initial grounding for developing key themes and included in-depth interviews (IDIs), focus group discussions (FGD) and unstructured observations at health facilities. In phase 2, further fieldwork was completed to review these themes and expand on earlier findings using IDIs and field notes of unstructured observations. Phase 1 interview guides were developed following extensive literature review and discussions amongst the research team, and followed a number of research themes including nature of work, feelings about work and work environment, reasons for being involved in work, strengths and limitations about current work and perception about the implementation of a MC programme for HIV prevention. Following the emergence of themes in phase 1, further questioning was incorporated in phase 2 to examine perception of service being delivered and experience and feelings towards working in the sexual and reproductive health field. The interview guides were revised on an ongoing basis during both phases to elicit more focused responses from participants and to accommodate themes that emerged in the early stages of data analysis. IDIs and FGDs were completed in TokPisin (a lingua franca of PNG) or English by trained fieldworkers in both phases. IDIs and FGDS were digitally recorded, transcribed verbatim and translated into English, where necessary, by a team of six researchers.

### Sampling

**Figure 2:**
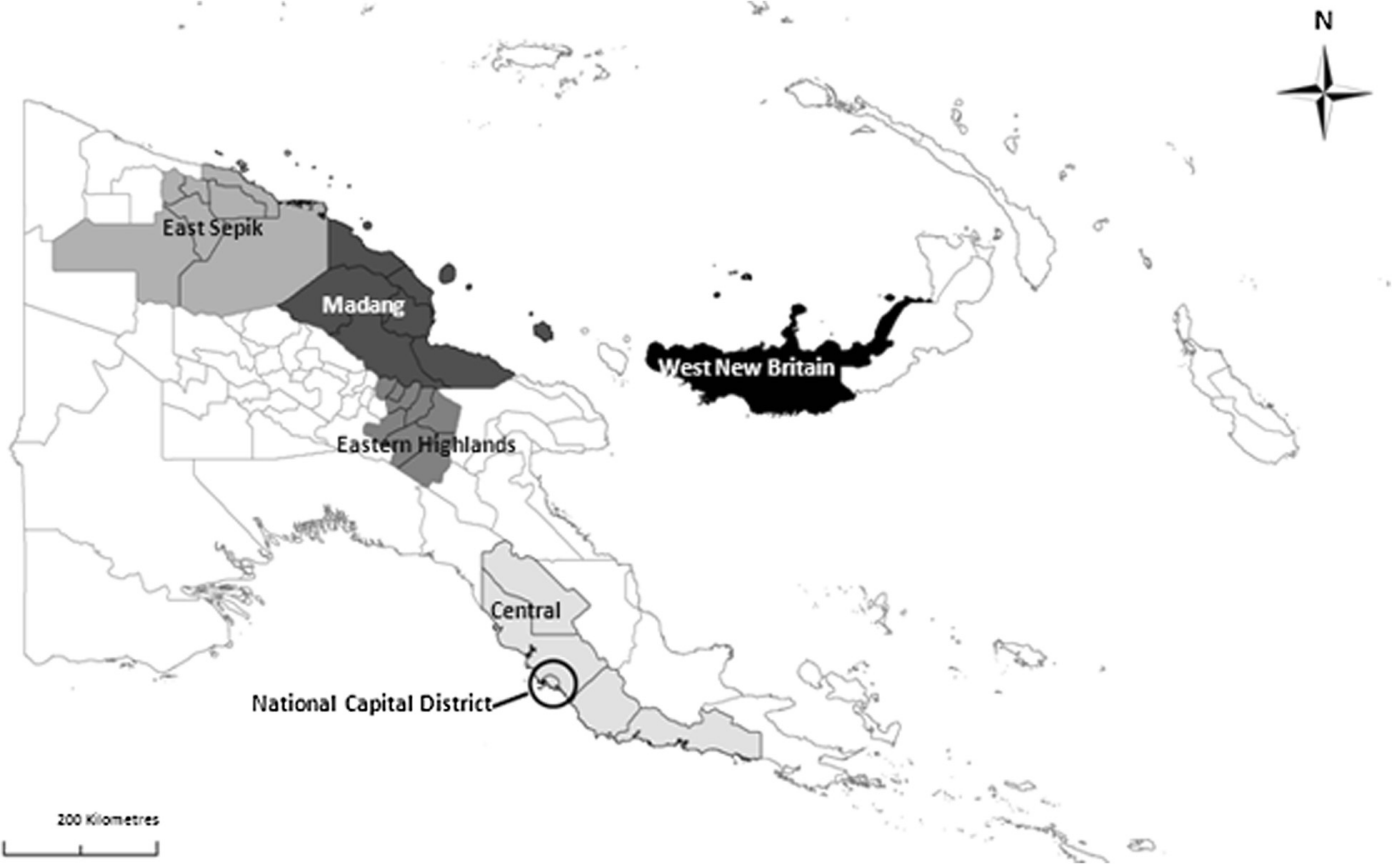
Map of Papua New Guinea with study provinces highlighted.

Participants were selected in collaboration with key national and local stakeholders at each study location using purposive sampling strategies based on the participants’ involvement in activities related to sexual and reproductive health, and potential for involvement in a future MC programme. The activities included NSV, HIV and AIDS care and treatment, sexually transmitted infection testing and treatment, and sexual and reproductive health counselling and education clinics. Participants included medical officers, nursing officers, health extension officers, CHWs, and support staff from health facilities in five provinces (NCD, West New Britain Province, East Sepik Province, Eastern Highlands Province (EHP) and Madang Province) (Figure 2).

Twenty-nine IDIs were conducted with frontline HWs in the five provinces over both phases (Table 1). One FGD was completed with three HWs due to limitations of time for the participants to complete individual interviews and the suggestion by other key informants that they would offer important insight into the research question. In phase 1, a total of 13 frontline HWs participated in the interviews. In phase 2, another 19 interviews were completed with three key frontline HWs re-interviewed due to them being active leaders in the area of sexual and reproductive health and therefore their involvement could provide additional insight into service changes since phase 1. In total, 17 men and 12 women took part, with the distribution of participants across the health-care profession and across gender listed in Table 2. Most participants had been working in their profession for over 5 years (Table 3). A total of 16 health facilities were represented, including provincial hospitals, outpatient clinics and rural health centres (Table 4).

**Table 1:** Data collection by study phase, type and province

**Phase 1**	**IDI**	**FGD**	**Phase 2**	**KII**	**FGD**
West New Britain	2	1	West New Britain	–	–
Eastern Highlands	2	–	Eastern Highlands	3	–
National Capital District	4	–	National Capital District	12	–
East Sepik	2	–	East Sepik	3	–
Madang	–	–	Madang	1	–
Total	10	1	Total	19	–

FGD, focus group discussion; IDI: in-depth interview.

**Table 2:** Distribution of participants across health profession

**Health worker**	**Females**	**Males**	**Total**
Medical officers	2	7	9
Nursing officers	6	1	7
Health extension officers	2	2	4
Community health workers	0	5	5
Other^a^	2	2	4
Total number of participants	12	17	29

^a^Includes volunteer, administrative and counselling staff.

**Table 3:** Years as a health worker across discipline

**Years in service**	**Medical officer**	**Nursing officers**	**Health extension officers**	**Community health workers**	**Other**	**Total participants**
<5	4	0	1	1	2	8
5 to 10	0	0	0	1	1	2
10 to 20	1	3	2	1	1	8
>20	4	4	1	2	0	11

**Table 4:** Data collection by study phase, type and province

**Setting**	**NCD**	**WNB**	**Madang**	**EHP**	**ESP**	**Total**
Provincial hospital	1	1	0	1	1	4
Urban government outpatient clinic	1	1	1	1	0	4
Urban NGO clinic	1	0	0	0	0	1
Urban government/FBO clinic	2	0	0	0	0	2
Urban church clinic	0	0	0	0	1	1
Rural health centre	0	1	0	1	0	2
Private	2	0	0	0	0	2
Total number of settings	7	3	1	3	2	16

EHP, Eastern Highlands Province; ESP, East Sepik Province; FBO: faith based organization,; NCD, National Capital District; NGO, nongovernmental organization; WNB, West New Britain Province.

### Data analysis

Qualitative thematic analysis of the transcripts and field notes was undertaken using a social constructivist approach. This approach is considered particularly suitable for applied qualitative research in health-care settings [21,58]. Transcribed and translated interviews were read several times for familiarization [26]. A codebook was developed and all interviews were double coded. In cases of discrepancy in coding, a third researcher coded the selected text in question. The phase 1 interviews were analysed by three in-country researchers at PNG Institute of Medical Research in Goroka with key themes and findings influencing the progression of analysis in phase 2. In phase 2, the first author subjected all data to further thematic analysis, organizing data around the main themes of the framework developed by Franco, Bennet and Kanfer (Figure 1), and entered into the qualitative software program MAXQDA (VERBI Software GmBH, Berlin, Germany). Qualitative data analysis focused on comparing and contrasting specific themes as they occurred in each interview on the meanings and experience of working as a HW in sexual and reproductive health services in PNG. The final coding was reviewed by the in-country researchers and emerging themes were discussed with all authors and adjusted where appropriate.

### Ethical considerations

Ethical approval was granted in PNG by the PNG Institute of Medical Research Internal Review Board, the PNG National AIDS Council Research Advisory Committee and the Medical Research Advisory Committee of the PNG National Department of Health. In Australia, ethics approval was provided by the Human Research Ethics Committees of the University of New South Wales and the University of Queensland. Written informed consent was sought from all individuals who participated in interviews or FGDs. Confidentiality was maintained in the data transcription process, which used pseudonyms to identify respondents.

## Results

A social constructionist analysis of the qualitative interviews identified a number of key individual-level and sociocultural determinants that motivated HWs to be involved in sexual and reproductive health services in PNG. The key motivations presented were: a sense of citizenship or community responsibility; and accomplishment in their work. These two factors tended to define how HWs perceived themselves in their work roles, which often had an impact on service sustainability and success, particularly in rural areas. This analysis examines the key motivations to work in sexual and reproductive health services in PNG and the implications this may have for a future MC programme if deemed appropriate. The results are presented according to the social and individual determinants of motivation as described by Franco, Bennet, Kanfer and Stubblebine (Figure 1).

### Social factors of motivation: sexual and reproductive health care in the context of PNG

#### Working in challenging environments

The sociocultural environment had a significant impact on the motivation of the HWs, including community expectations and social values as described in Figure 1[40]. All of the HWs perceived themselves as front-line workers in a dangerous and contested environment, with perseverance despite these challenges being a defining characteristic particularly in Port Moresby, NCD. Port Moresby, the capital city of PNG, is well known for its violent crime that threatens the safety and security of all citizens. For example, one clinic visited in Port Moresby had recently moved to a new location due to problems with armed robberies and other security issues. The need to have security guards at entrance gates was common at most health facilities visited, but particularly in urban settings.

The risk to physical safety taken by staff, including volunteers, who enter volatile areas, such as settlement areas, market places and villages, to assist in the follow-up of clients and in health promotion activities was highlighted by many respondents. While risk is most obviously associated with Port Moresby, risk was also identified in other contexts including the associated risks of travelling for outreach service such as poor roads, weather and other roadside crimes. For example, in the mountainous area of the highlands of EHP, poor road conditions are a frequent hazard along with unpredictability of civil unrest amongst neighbouring tribes. The best illustration of other forms of physical risk is explained by a medical officer in NCD:

They [the volunteers] do all the hard work in the field. They get abused for talking about sex openly in the market and in the villages. They actually have been threatened and some of them have even had their bags stolen. But they put up with all of this. (Male medical officer, NCD)

Social risk, or the risks associated with the stigma of working in sexual and reproductive health, was also a concern for many. Dealing explicitly with matters pertaining to sexual and reproductive health is largely seen as a cultural taboo by the whole of the PNG community:

This is a Melanesian society and they will criticise you … In our culture we don’t go around going into details about human sexual health, but we are working through this. So this topic [sexual health] is often seen as shameful to our clients, other community members and other people in our country. (Female medical officer, NCD)

However, although all the HWs acknowledged the risks, both physical and social, they all were able to describe other positive inspiration for engaging in these health services.

#### Community relations

The local community played an integral role in the performance and motivation of most of the HWs, with positive motivation more likely to arise from interactions with the community rather than from the health system itself. Most of the HWs expressed dissatisfaction with the administration of the health system, arguing that this also contributed to the difficult working environment. For example, according to some HWs, key officials rarely acknowledged or showed appreciation for the work that they did. However, as one CHW described about his involvement in a sexual and reproductive health programme, despite this negativity there were other reasons that inspired service.

… I think I am doing a lot of work that the government doesn’t see, and at times I feel I want to leave this job and go away. But, the heart that I have for my own men is why I have been so patient. So I am still doing this work. (Male community health worker, EHP)

For most respondents, the sense of community was local and specific and inspired a deep commitment to the work that was being done. A few respondents were motivated by the perceived contribution the service had to improving the socioeconomic environment of the community. For example, one HW’s own perceived assumption that men required vasectomy due to the economic pressures of having a larger family in PNG was the stated impetus for his involvement in NSV. By providing the NSV service the HW would be alleviating men and families of these pressures, particularly in more rural areas.

The success of the service is because the people are in need of the service. I think they are considering their needs like school fees, food and cooking. So, they tell me that to control their family, it will make their lives easier and they contact the service so they don’t have any more children and ongoing school fees etc. (Male community health worker, Madang Province)

A majority of the HWs were also supportive of a MC programme to be implemented in PNG. This support seemed to be also driven from their community concern, ranging from prevention of HIV to minimization of complications following penile foreskin cutting completed by a non-HW. For example, as one respondent explained:

I will definitely encourage people to come for circumcision because it’s healthy, and like in PNG, most circumcisions are done in the village and it is dangerous. (Female nurse, West New Britain Province)

However, one HW was resistant about the possibility of a national MC programme for fear that this would raise community expectations of complete protection from HIV and could result in retribution in the event of a circumcised man becoming HIV-positive. The complex network of cultural obligations in which HWs engage as a result of the cultural context of the communities in which they work was also identified to contribute to HW engagement in programmes:

If circumcised men are infected, they will point their fingers to us the health people and say, ‘You said I will not get it and I went for circumcision but now I got it and your words are lies’. They will not believe us and this has its consequences too. (Male health extension officer, EHP)

HWs perceived themselves as actors, able to effect local change. The importance that a HW placed on their service heightened their determination to succeed. In some cases, HWs demonstrated particular concern and urgency to deliver services that may be considered elective or non-essential.

They have a problem in the village and I need to help, I need to stay with them and perform vasectomies in the villages. It’s my heartfelt sympathy for my own people. (Male nursing officer, NCD)

This sense of community was also articulated at a national level by a few respondents. Despite the social diversity of the respondents and known existence of significant tribal divisions in PNG, some comments were framed in terms of national pride and a genuine concern for the ‘fellow countrymen’ they served. This depth of emotion was evident in the narrative of one respondent, who wept as she reported:

All the things we do are for Papua New Guinea people. Just to serve the Papua New Guinea people is an honour. This [the work done in the clinic] is very important, for my country and for my people. I can work the extra hours or extra weekends. If I have to serve it I will serve it. I do have a lot of commitment for this project. I am the back bone of this project and I do all the background. You know, I will do it for the name of Papua New Guinea. I want to serve my people. So if it means taking a weekend then I will do it. I have a heart for my people. (Female administration officer urban clinic, NCD)

This sense of commitment to serve the country appears to be best explained as being embedded in the religious convictions of the HWs.

### Service, sacrifice and religious conviction

Sacrifice, humility and willingness to extend the boundaries of their roles was an attribute that most respondents described as important for HWs to be successful in delivering sexual and reproductive health programmes. Acknowledging the constraints in health service delivery in general in PNG, many saw the need to do whatever was possible.

To help the local people and the people of Papua New Guinea, we just have to serve our people with what little we have and manage it. (Female nursing officer, NCD)

Many frontline HWs expressed how their own dedication and perseverance resulted in programme and service success. Some frontline HWs also reported self-funding parts of the service, such as travel, accommodation and health promotion, if this was needed. They felt it was often difficult to maintain momentum given system difficulties such as obtaining funding or access to transport. Being flexible to the constraints was often needed.

I went through kind of a stressful time. And I was thinking, if I don’t get clients, then what I was trained to for is, useless … So I used a lot of my personal money. You had to, to get fuel, hire a vehicle and buy refreshments. It was quite a great challenge to me. But I did it. (Male nursing officer, NCD)

The commitment to serve appeared to be underpinned by the influence of religion, with HWs valuing ‘doing good’ as an important motivator for their work. The role of religious faith and a commitment to serve was a key theme identified throughout all interviews and played an important role in the impetus to work within sexual and reproductive health. The perception that God had made it possible for them to succeed was directly acknowledged as an important motivator for a few.

The big man [God] gave me this idea and … I was proud because I have never gone to a school for this … And I thank the big man from above [God] who is giving me more wisdom and knowledge for this work. Thank you. (Male community health worker, East Sepik Province)

### Individual-level determinants of motivation

#### A sense of purpose

Individual characteristics that seemed to be common amongst all HWs interviewed included: flexibility and sacrifice; a sense of achievement and recognition for work; and strong determination for success despite the barriers and constraints to service delivery within the health system. Feelings of responsibility and a desire to improve people’s health were frequently reported as having attracted respondents to join the sexual and reproductive health workforce in PNG. A strong sense of purpose and passion for the work that they did further assisted in mitigating some of the pressures and challenges of implementing sexual and reproductive health services in the country. This genuine commitment and a deep belief in their responsibilities fuelled individual-level determinants of motivation.

I have got big interest in my job, I influence myself [to work in sexual health and health promotion] because I believe that I have to teach others to be healthy. (Male community health worker, East Sepik Province)

A strong sense of purpose intensified a genuine passion for the work that was being done by all levels of HWs interviewed. An unofficial but deeply important requirement for joining the sexual and reproductive health workforce as evident in the data included determination and a deep regard for the work that they did.

It is something that I am enjoying so us health workers need to do this job [vasectomy service] … I fell in love with this project. (Male nursing officer, NCD)

A sense of purpose was not only inspired by the sociocultural environment of the communities they served, as described above, but also how they viewed the uniqueness of their service. A few HWs observed that their service was unable to be delivered by anyone else, which further justified and inspired their commitment.

So from our sacrifice, and hard work, you can see that the vasectomy program in this province has grown so much. When there is a lot of interest in men, even though we have these problems [issues with funding and transport] we still carry on. Despite the problems, we still go into the villages to do vasectomy. Because there is no one … there is no one apart from this team. (Male community health worker, EHP)

#### Achievement and recognition of work

Perceptions of how other health organizations viewed the HWs’ facility and service also inspired motivation. HWs often expressed feelings of pride towards what their clinic or service was doing.

To me I am happy. I am happy and proud because our organisation assists with identifying what is happening here so it is globally known. So it motivates me and whenever anyone comes for interview or anybody comes to see my clinic I am always there for them because of this. (Female nursing officer, NCD)

Some HWs, particularly CHWs, also showed considerable satisfaction in the achievement of being able to master a skill such as NSV. This was heightened by the fact that many other CHW colleagues had not been specifically trained and therefore did not have these higher level clinical skills. This was observed by both upper health system workers as well as frontline HWs.

I think the community health worker group they are sort of very interested [in learning NSV skills] the male ones, because it is like an added skill; an extra skill that they are going to have. And so there are a lot of them that are interested to be trained. (Male medical officer and programme coordinator, NCD)

CHWs were particularly considered by upper health system officials to have stronger connections to the community than higher level HWs, which strengthened service sustainability. For the NSV programme, CHWs were considered more successful than doctors in being able to implement the programme in their communities. However, with additional skills such as NSV, the CHWs also showed some dissatisfaction with not being recognized financially.

Every community health worker has the same salary. But this vasectomy is only performed by doctors and HEOs [health extension officers] and specially trained CHWs … He [another CHW] has been performing vasectomies since 2002 and has never been paid differently. He has been performing vasectomy but is still paid on a typical community health worker salary. The same. Nothing different! (Male community health worker, EHP)

Although most HWs were resolved to the challenges of the working context, many considered that the importance of their work needed to be recognized. The suggested recognition typically included increased wages or other financial incentives.

## Discussion

With such tremendous cultural diversity and evident complexity of HW involvement in penile cutting activities, innovative strategies are required to conduct an efficient MC programme [15-59]. Understanding HWs’ relationships to the health system, resources and environment in which they operate and the impact this has on performance and motivation has key implications for a potential MC programme. HWs reported service delivery barriers working within PNG that undermine motivation, including issues around security. However, those interviewed expressed a genuine resolve to rise above these constraints and uphold the sexual and reproductive health services they were delivering. The impact of community influence and expectation, religious conviction and the role of different incentives need to be considered when introducing a MC programme, particularly to communities where penile foreskin cutting is a common and accepted practice. This study has presented some of the sociocultural and individual determinants that affect motivation among sexual and reproductive HWs in five provinces in PNG.

### Implications for delivery of an adult male circumcision programme in the context of PNG

#### Religious convictions and the concept of service

The religious convictions evident in HWs will have potential complications with the delivery of any sexual and reproductive health service, particularly one such as MC that to some degree may relate to or contradict religious practice. Religious convictions are motivational determinants that have not been well described in studies of HW motivation in other countries. Religious affiliations and the commitment to serve have been shown to have significant individual-level impacts on HW motivation in PNG [18,19,47,49]. The religious undertones of commitment were also described by respondents in this study, confirming the strong impact that the social values and beliefs of HWs and the communities they service have on their performance [47,49]. Although it was at times difficult to distinguish between what may have also been general health professional values, motivation was certainly deeply embedded in community expectations and social values.

#### Community relations, expectations and perceived responsibilities for the state of the health system

Individual differences are relatively enduring characteristics of the individual, but others are formed by the acculturation of the individual within the larger societal context [40]. Health system failures in PNG have resulted in complications in securing funds; in supplying, training and supervising staff; in accessing equipment and medicines; and in governance and leadership [14,16,17,60,61]. In the case of sexual and reproductive health services in PNG, the results of this study indicate that individual HWs tended to assume responsibility for this failed system. This is a similar finding to Mbilinyi and colleagues in Tanzania, where HWs were forced to take responsibility for dealing with problems arising from organizational inefficiencies [26]. HWs in PNG adapted to a dysfunctional system by adjusting service to compensate for key barriers to service delivery, acknowledging a commitment to be flexible an important attribute. This was also an important attribute for the challenges HWs reported. This included the general safety and security issues of work and travel in PNG, the associated stigma of work that dealt with matters pertaining to sexual health, and the potential burden on the individual HW to provide accurate and effective service to others within their communities.

Trust, recognition and appreciation from the community can enhance the ability and willingness of HWs to provide an efficient service and have been shown to be integral to HW motivation in a number of other studies [23]. In this study, HW links to the community appeared stronger than their links to the organization, resulting in community expectation and peer pressure having a much stronger impact on HW motivation than organizational influences, as reported in earlier research from PNG [18,20,53]. As a result, competence was seen to be more valued from a community-building perspective rather than in terms of organizational capacity. In some cases, this may be the result of isolation of the service or HW, but also seems to be a part of the culture of service and social responsibilities of the HW in PNG.

Community expectations may impact on HWs’ willingness to take on a programme, or may commit HWs to be directed by the high demand for the service in the community. These obligations may generate unwillingness of HWs to participate in programmes such as MC if there is a chance of community misperception. Obligation to the community may also commit the HW to perform specific types of foreskin cuts determined by the community and not the health system. For example, previous research have shown the preference for longitudinal foreskin cuts or dorsal slit within PNG communities over the circumferential cut, which the African trials utilized [7,9]. Establishing a strong link of support from the health organization to the individual HW is important to facilitate better outcomes and high-quality, standardized service delivery. Further understanding of the potential social pressures to which HWs may be exposed in PNG is critical. Given the heightened awareness of MC for HIV and the demand for penile cutting already evident, HWs are likely to be faced with complex demands. Without the support of a resilient health system, the social factors of community expectations and social responsibility of the HW are likely to take precedence.

#### Task shifting and other incentives

HWs reported performing additional duties from technical to administrative tasks beyond their job specification, with additional technical duties more likely to be taken up by rural HWs. This study confirmed the results of earlier research in PNG that showed HWs saw positive opportunities in enhancing their skills and other personal development, which compensated in part for needing to fill the gaps created by understaffing [18]. Pride in accomplishing a new skill was particularly important to HWs in more remote areas and among those who were acquiring skills different to those of their colleagues and peers. This finding further demonstrates the influence of other non-monetary factors of motivation.

Although financial rewards were not indicated by many of the respondents as integral to motivation, many still saw the importance of recognition for their work. Other studies have also reported on the lack of appreciation and limited feedback from supervisors as a demotivator [62]. However, while financial rewards have been widely discussed as motivational levers, they should be used with a combination of strategies based on country-specific needs and application [42]. Improving motivation to perform well will require multiple interventions and should be integrated with other incentives and interventions such as improved infrastructure to create a more balanced approach to increase motivation, satisfaction and performance [20,40,63].

The results also suggest that the strong ties CHWs have to the community may be beneficial for to implementation of any new programme. However, if task shifting was to occur and CHWs were to be trained in MC, this has potential medico-legal implications and, unless explicitly covered by regulation, may affect future role descriptions, reporting relationships and remuneration. Requiring CHWs to develop complex new skills, while maintaining their current pay rate, may result in dissatisfaction and be demotivating. Recognizing that HWs value opportunities to increase skills and responsibilities also has implications for a MC programme [18-20].

### Limitations of study

The study focused on a variety of health facilities that may be called upon to implement a future national MC programme. This is reflected in the range of duties, challenges and workload of health providers and also their need to be self-sufficient and have a broad skill base. Generalization of the results beyond the sites visited may be difficult given the qualitative nature of the study and the probable differences in sociocultural contexts compared with other provinces and regions in PNG. However, the issues emerging reflect phenomena relevant for policy considerations around a MC intervention or other proposed complex sexual and reproductive health intervention.

Another potential criticism is that the selection of participants was based on their demonstrated willingness to cooperate with the research team, with resultant potential bias towards more motivated participants. However, participants consisted of a number of different professionals from a number of different facilities in a number of different areas. While they may display more positive attributes of motivation than other members of the general workforce, their responses were useful in terms of the range of factors influencing motivation. It was also difficult to explore the motivation of HWs to deliver a MC programme explicitly due to the varied experience and awareness of the HWs for MC as a HIV prevention strategy.

## Conclusion

The results of this study confirm that motivation is not a function of a single determinant, but is the result of a complex interplay of factors that operate within a cultural context. An effective MC programme for HIV prevention must therefore operate on the set of key determinants, and will need to address local contextual factors as well as broader health sectorial factors that are affecting worker motivation at the local level [40]. Certain similarities among key determinants exist between other developing and transitional countries, including, pride, organizational support, job properties and values [40]. However, the interconnectedness of the HW with the community appeared to be unique to PNG compared with what other studies have discussed. Introduction of new health programmes such as a MC programme for HIV prevention is likely to impact upon one or more of the many motivational determinants, and those engaged in development of policy need to consider carefully such impacts prior to implementation. These findings highlight the need to take into account the differing needs of communities as well as the motivational influences on HWs that exist within the sociocultural environment – in particular, consideration of the local workforce and culture, and the potential impact this may have on delivering a service such as MC that already may have significant demand and meaning within some communities. If a national MC programme is to be implemented in PNG, failure to acknowledge the impact of HW motivation to perform and engage in such a programme within policy discussions could significantly limit future programme success.

## Abbreviations

CHW: community health worker; EHP: Eastern Highlands province; FGD: Focus group discussion; HW: Health worker; IDI: In-depth interview; MC: Male circumcision; NCD: National Capital District; NSV: Non-scalpel vasectomy; PNG: Papua New Guinea

## Competing interests

The authors declare that they have no competing interests.

## Authors’ contributions

AT & PSH, participated in the conception of study design with feedback and guidance from AV & AK. The field research activities were supported by AK, AT, MK, JN, HA and RN. Data analysis and manuscript drafting was carried out by AT and PSH with support and contributions from AV, AK, PS, GL, MK, HA, JK, JM and PS. All authors read and approved the final manuscript.
